# Implementing a Multimodal Palliative Care Curriculum: Impact on Knowledge, Confidence, and Skills

**DOI:** 10.5811/westjem.53884

**Published:** 2026-06-28

**Authors:** Travis Hase, Cindy Ndiaye, Margaret Putman

**Affiliations:** * Advocate Christ Medical Center, Department of Emergency Medicine, Oak Lawn, Illinois; † University of Illinois College of Medicine at Chicago, Department of Emergency Medicine, Chicago, Illinois; ‡ Wake Forest University School of Medicine, Department of Emergency Medicine, Winston-Salem, North Carolina; § Advocate Christ Medical Center, Department of Graduate Medical Education Research, Oak Lawn, Illinois; || University of Chicago Pritzker School of Medicine, Section of Geriatrics and Palliative Medicine, Chicago, Illinois

## Abstract

**Introduction:**

Emergency physicians frequently care for patients with serious or terminal illnesses, yet they often lack formal palliative care training. Our primary objective was to develop a structured, multimodal palliative care curriculum for emergency medicine (EM) residents and evaluate whether this curriculum improved residents’ knowledge, comfort level, and perceived application of skills to care for patients with chronic or terminal illness in the emergency department (ED). Our secondary objective was to determine whether EM residents found palliative care education important and to identify which educational modalities are most effective for acquiring palliative care knowledge and skills.

**Methods:**

We implemented an eight-hour multimodal curriculum for EM residents at a single, large Level I trauma center (four hours of didactics, a three-hour simulated patient communication skills lab, and one hour of high-fidelity simulation). Our primary outcome was pre- and post-intervention surveys (12 questions) that assessed perceived knowledge, comfort, and skill application on five-point Likert scales. We analyzed paired responses using the Wilcoxon signed-rank test, with a *P* value of < .05 considered statistically significant. Effect size was calculated using Cohen *d*. Our secondary outcome measure was a post-intervention survey (six questions) that assessed participants’ opinions on the effectiveness of the different educational methods.

**Results:**

There was a 100% response rate among 41 residents from all postgraduate years 1–3. Significant improvements (*P* < .001) were observed in residents’ self-reported abilities across all domains with large effect sizes. Median scores and interquartile ranges increased for conducting goals-of-care discussions (4 [3–4] vs 4 [4–5]), interpreting advance directives (3 [2–4] vs 4 [4–4]), managing end-of-life symptoms (3 [2–3] vs 4 [3–4]), communicating bad news (3 [2–4] vs 4 [4–4]), and coordinating with palliative or hospice teams (2 [2–3] v. 4 [4–4]). All educational modalities were rated effective, with simulation and small-group sessions preferred over lectures.

**Conclusion:**

A structured, multimodal palliative care curriculum significantly enhanced EM residents’ perceived preparedness to manage patients with palliative care needs. Embedding didactic and simulation-based palliative training in EM residencies is both feasible and impactful, addressing critical gaps in palliative competencies and aligning with national best-practice guidelines.

## INTRODUCTION

### Background

As the U.S. population ages and becomes increasingly medically complex, the need for palliative care far exceeds the availability of subspecialty-trained palliative care physicians.^1^ The emergency department (ED) remains a frequent point of contact for these patients. Half of those living in the United States visit the ED in their last month of life and 75% in the prior six months, with frequent repeat visits.^2^ Most adults do not have advance directives completed at the time of ED visits.^3^

Because many patients present to the ED while critically ill and requiring urgent interventions, it is imperative that emergency physicians be able to quickly align with patients and families to determine values and offer recommendations for further medical treatment. A systematic review completed in 2019 found that palliative care in the ED (most often delivered by palliative care specialists) improved quality of life, reduced hospital length of stay, and did not decrease survival rate compared with usual care.^4^–^5^ Having early and effective goals-of-care conversations can improve symptoms, reduce suffering, and decrease healthcare costs.^6^–^9^ Implementing palliative care in the ED for actively dying patients has been shown to improve patient care, optimize comfort measures and emotional support, increase patient satisfaction, and reduce overall hospital admissions.^10^ Emergency physicians often practice in environments without readily available palliative care specialists and, therefore, need to have primary palliative skills to provide excellent and holistic care to patients with chronic and/or terminal illnesses.^11^

Emergency physicians have reported that palliative care is an important competence in their practice, yet they feel they are not adequately educated in providing such care. They report discomfort regarding physician orders for life-sustaining treatment forms and navigating conflict around withholding life-prolonging treatment.^12^–^16^ A needs assessment of emergency medicine (EM) residents showed specific areas in which there were self-reported knowledge gaps, including managing hospice patients, withdrawal of life support, prognostication, and pain management.^17^

Palliative care training in EM residency is highly variable. One study that surveyed EM residency program directors showed a general lack of formal palliative care training in EM residencies, with opportunities for competency training and gaps in resident skill level.^18^ Previously reported EM residency palliative curricula have included adaptation of the Education in Palliative and End-of-Life Care Emergency Medicine (EPEC-EM) curriculum in synchronous and asynchronous formats, as well as case-based simulation, and were effective methods to increase knowledge and increase resident confidence in palliative care skills.^19^–^20^

In 2018, an expert panel assembled by the American College of Emergency Physicians Palliative Medicine Section, consisting of academic EM faculty, community emergency physicians, EM residents, and nurses, created hospice and palliative medicine EM milestones. These competencies are modeled after the EM milestones established by the Accreditation Council for Graduate Medical Education and can serve as guidance for EM residency programs.^21^ In 2021, best-practice guidelines for primary palliative care in the ED were published, again emphasizing the importance of this skillset for emergency physicians.^11^

**Population Health Research Capsule**

What do we already know about this issue?

*Emergency physicians often feel ill-prepared to address palliative care needs in the ED, yet palliative care training during residency remains limited*.

What was the research question?

*We investigated whether a multimodal curriculum would improve residents’ comfort with and knowledge of palliative care skills*.

What was the major finding of the study?

*Participants reported significant knowledge improvements across multiple domains of primary palliative care (P <.001 for all domains)*.

How does this improve population health?

*Integrating palliative care training in EM residency may improve outcomes for patients with life-limiting illness. Further research is needed to assess translational impacts*.

### Study Objectives

Our primary objective was to develop and implement a multimodal palliative care curriculum for EM residents at a large, tertiary center and to evaluate its impact on residents’ knowledge, level of comfort, and perceived skill application in caring for patients with serious life-limiting and/or terminal illness in the ED. A secondary objective was to determine whether EM residents find palliative care education important and to identify which modalities they found effective to acquire palliative care knowledge and skills.

## METHODS

### Curriculum Design

In our review of the literature, previous palliative care curricula for EM residents have predominantly employed single educational modalities, most commonly didactics or a single simulation experience. We identified an opportunity to provide a more comprehensive and layered simulation approach that combines small-group simulated patient workshops with high-fidelity manikin simulation, allowing for deliberate practice and scaffolded learning.

This was a pilot curriculum with a goal of determining effectiveness, feasibility, and durability within our institution. Our intention was to create a framework that would be easy to replicate and adapt to future needs. We sought to use educational modalities that optimized the benefits of experiential and simulation-based learning. Curriculum implementation followed Kern’s standardized six-step curriculum design process.^22^ We conducted a general needs assessment by reviewing available published survey data of emergency physicians, residents, and program leaders, and we identified knowledge and experience gaps in several domains specific to palliative care. A targeted needs assessment of our residents was then performed to focus our intervention’s efforts on the domains our learners felt they would encounter in their practice.

With this information, we developed a palliative care curriculum for EM residents. Total training time was eight hours. The curriculum consisted of four didactic sessions, each lasting one hour; a three-hour communication skills lab using simulated patients; and an hour of small-group simulation using a high-fidelity manikin. The format of the simulation case necessitated the active participation of all learners. This pilot curriculum was implemented over a six-month period during regularly scheduled conference and simulation learning times.

The first didactic session was an introduction to palliative care. In this session, palliative care was defined and differentiated from hospice care, learners were taught to interpret various advance directive forms, and to understand the role that emergency physicians may play in the overall care of patients with serious life-limiting or terminal illnesses. The second didactic session focused broadly on the principles of palliating symptoms, ensuring comfort, and addressing symptoms refractory to usual care. It also included teaching on disposition options for hospice and patients with palliative needs. The third didactic session focused on teaching about the active death process, end-of-life care, and transition to hospice. In advance of the communication skills lab, a fourth didactic session focused on providing a toolbox of communication skills and techniques for assessing values, communicating bad news, and assessing goals of care as part of a serious-illness conversation. Specifically, several well-developed communication tools were reviewed, including the SPIKES protocol for delivering bad news, the NURSE mnemonic for responding to emotion, and the REMAP framework for goals-of-care conversations.^23^–^26^

The communication skills lab focused on providing learners with the tools to hold a rapid goals-of-care conversation with patients and families. Frameworks for delivering bad news, assessing values, and providing values-based recommendations were taught. Learners then had the opportunity to practice these skills in small groups, rotating through six different scenarios with simulated patients in a rapid-cycle deliberate practice format.^27^ Lastly, learners participated in a small-group, high-fidelity simulated patient experience where they were asked to apply the skills they had previously learned. Specifically, they evaluated a patient with terminal and worsening illness, delivered bad news based on the findings of their workup, assessed the patient’s values, and transitioned into a goals-of-care conversation. Learners needed to discuss palliation and medical management options and ultimately provide recommendations based on an alignment with the patient’s values.

### Study Design

Approval was obtained from the institutional review board, and the study was deemed exempt. We performed a comprehensive literature review of palliative care needs in the ED and previously implemented palliative curricula for EM residents. The study population was EM residents in all levels of training at a large, university-affiliated Level 1 trauma and tertiary referral center. All residents were exposed to this curriculum regardless of participation in the study; completion of the surveys was voluntary. Inclusion criteria were all residents who completed both the pre- and post-intervention surveys. One resident was excluded from the study population, as they were a co-investigator.

Surveys used Likert-scale questions to capture residents’ perceived ability regarding each component. This survey instrument was created de novo based on identified needs from the published literature and consisted of 12 identical questions on both the pre- and post-survey.^11^, ^17^, ^18^, ^21^ In addition to a self-assessment, participants were asked six questions on the post-survey to evaluate the effectiveness of the various educational modalities used in our curriculum: didactics; small-group learning with simulated patients; and simulation-based experiential learning. As simulation training likely poses the greatest barrier to replication due to cost, equipment availability, and faculty experience, our survey asked additional questions regarding simulation to inform and support future resource allocation.

The pre-intervention survey data were collected from July 26–August 2, 2022. Post-intervention survey data were collected from December 21, 2022–January 1, 2023. We sent pre-notifications and targeted reminders during the survey collection window to achieve a high response rate.^28^ No incentive was offered for survey completion.

### Research Methods

To conduct a statistical comparison, we converted Likert-scale items to corresponding numerical values (1–5): strongly disagree; disagree; neutral; agree, and strongly agree. For each Likert-scale question, their distribution was assessed for normality via the Shapiro-Wilk test (*P* > .05 denoting a normal distribution). All variables were found to be not normally distributed, leading to the use of the nonparametric equivalent of the paired *t*-test, the Wilcoxon signed-rank test. A *P* value of < .05 determined a significant difference in Likert scores pre-tests versus post-tests. We calculated effect sizes for each survey item using Cohen *d*. And we used the statistical software SAS v9.4 (SAS Institute Inc, Cary, NC) for all statistical analyses.

## RESULTS

There was a 100% response rate, with 41 of 41 potential respondents across all postgraduate years (PGY) fully participating. In total, 13 PGY-3, 14 PGY-2, and 14 PGY-1 residents engaged with the curriculum and completed both the pre- and post-intervention surveys in their entirety. Table 1 details the survey questions and the median Likert-scale survey score with interquartile range, both pre- and post-intervention, as well as the effect size for each question.

Survey results showed most residents agreed (36.6%) or strongly agreed (51.2%) that EM has an important role in providing palliative care. Most residents agreed (48.8%) or strongly agreed (41.5%) that palliative care education was important in EM residency training. There was no significant change in these opinions pre- versus post-implementation (*P* = 1.0 and *P* = .83, respectively). There was a statistically significant improvement in residents’ understanding of the differences between hospice and palliative care (*P* < .001).

All other survey questions assessing perceived confidence in knowledge and skills pertaining to palliative care were found to have statistically significant improvement pre- versus post-intervention, with large effect sizes (Table 1). These items evaluated ability to determine decision-making capacity, have goals-of-care discussions, interpret advance directive forms, treat symptoms associated with the last hours of living, effectively negotiate decision making with patients and families, treat refractory symptoms associated with chronic or terminal disease, perform effective acute pain management in patients with palliative care needs, and effectively communicate bad news. Residents’ ability to initiate contact with and involve palliative and/or hospice teams to optimize care for patients in the ED also had statistically significant improvement (*P* < .001).

**Figure 1:**
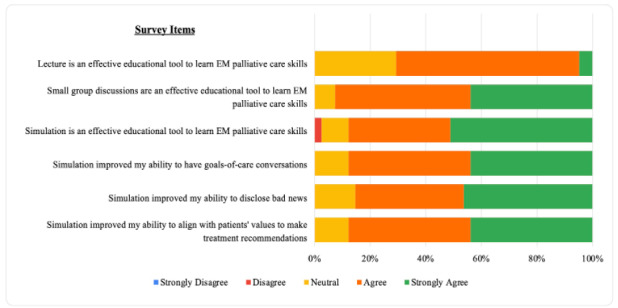
Survey results of emergency medicine residents’ preferred educational modality to learn palliative care skills. *EM*, emergency medicine.

Survey results evaluating learning modalities showed residents felt lecture, small-group discussion, and simulation were all effective educational tools (Figure 1).

The prompt “Lecture is an effective educational tool to learn EM palliative care skills,” resulted in 29.27% neutral, 65.85% agree, and 4.88% strongly agree. The prompt “Small-group discussions are an effective educational tool to learn EM palliative care skills,” resulted in 7.32% neutral, 48.78% agree, and 43.9% strongly agree. The prompt “Simulation is an effective educational tool to learn EM palliative care skills,” resulted in 9.76% neutral, 36.59% agree, and 51.22% strongly agree; 2.44% of residents disagreed about simulation’s effectiveness.

Regarding simulation specifically, 12.20% of residents felt neutral, 43.90% agreed, and 43.90% strongly agreed that simulation improved their ability to hold goals-of-care conversations, while 14.63% felt neutral, 39.02% agreed, and 46.34% strongly agreed that simulation improved their ability to disclose bad news. Regarding the perception that simulation improved their ability to align with patients’ values to make treatment recommendations, 12.20% felt neutral, 43.90% agreed, and 43.90% strongly agreed.

## DISCUSSION

The implementation of this palliative care curriculum for EM residents demonstrated a significant improvement in their perceived knowledge and skills across multiple domains of palliative care. Consistent with expected, early-stage outcomes following a curriculum intervention, the self-assessment results reflect perceived improvements in key palliative care competencies, aligning with the “knows” and “knows how” levels of Miller’s pyramid.^22^ These findings represent gains in learner confidence and self-reported preparedness and are best understood as necessary precursors to subsequent behavioral change and skill acquisition, which warrant evaluation through objective, performance-based assessments in future work. The structured, case-based approach provided residents with opportunities for active learning, hands-on practice, and real-time feedback, all of which contributed to their development. Additionally, the integration of simulation-based training allowed residents to apply their new knowledge in a controlled setting. The ability of simulation to offer a high-fidelity, low-stakes environment for practicing skills makes it an ideal tool for learning sensitive and challenging communication skills.

Our results demonstrate that the deployment of a multimodal curriculum may be additive in addressing the knowledge and experience gaps in emergency physicians’ comfort and skills in managing patients with palliative, hospice, or end-of-life needs while in the ED. This work contributes to a limited body of research similarly addressing the gap in primary palliative care training for EM residents. Nguyen and Matese assessed the effect of a curriculum on self-perceived skill and knowledge acquisition and primarily used a self-directed learning format consisting of free, open-access medical education content along with a lecture series.^29^ Goldonowicz and colleagues used a single educational modality with a simulation case.^20^ Their format allowed for experiential learning from a limited number of study participants with most learners observing the simulation rather than actively participating. Benesch’s study group describes a longitudinal and multimodal educational intervention that used the fee-based VitalTalk training program for emergency physicians, EMTalk, and the RE-AIM framework to evaluate impact.^30^

Our work describes a comprehensive, multimodal curriculum consisting of varied educational methodologies. We leveraged the benefits of experiential learning using both standardized patients and high-fidelity simulation. Our simulation-based learning used rapid-cycle deliberate practice formats of practice, and the small-group, high-fidelity simulation case necessitated the active participation of all learners. Our didactics and communications skills lab are adaptable to evolving educational need, learner interest, and new methods. Notably, our content and overall methods avoid the use of proprietary courses and materials, because those courses may be cost prohibitive for some institutions.

Given this pilot’s success, we incorporated this palliative care training as an enduring and longitudinal component of our overall residency curriculum. We believe the time and financial investment to be acceptable and sustainable, especially for programs with a well-established simulation training program. The multimodal framework is easy to replicate, and specific content areas are sufficiently modifiable based upon evolving program-specific needs. Anticipated annualized cost of the curriculum is estimated to be approximately $2,000 and represents the expense of standardized patients and guest speaker honorarium. The capital, operating, and personnel expense otherwise associated with our existing simulation center and program are incurred irrespective of this curriculum.

Opportunities for future scholarship include an objective assessment of at least a subset of palliative care domains to assess knowledge and successful application of learned skills. Querying longitudinal attitudes toward palliative care in the ED, as well as gauging comfort in knowledge and skills after a longer interval, may also be insightful. Additionally, the future development of a mastery-learning curriculum for a subset of skills, in particular serious-illness conversation, symptom management at the end of life, and palliative extubation, may prove particularly useful in preparing trainees to skillfully approach sensitive conversations and complex symptoms with patients and their families.

## LIMITATIONS

Our study has several limitations worth discussing. While our high response rate limits non-response bias, our results are limited to a single institution and may not generalize broadly because of selection bias inherent to our study design. Furthermore, our results are limited by the lack of objective assessment from an outside observer and may not fully reflect skill acquisition. The assessment instrument used was a subjective, unvalidated questionnaire, which is vulnerable to the biases inherent in self-assessment. Self-assessment relies on perceptions, which may not accurately reflect actual skill and knowledge and is subject to response bias and the Hawthorne effect. Our educational modality survey is not symmetric across the varying domains; therefore, it is underpowered by design, limiting interpretability.

While an improvement in perceived abilities is not unsurprising after the implementation of a curriculum intervention, our study may be affected by a ceiling effect bias, whereby the post-intervention survey is limited in its ability to measure improvement. In the pre-intervention survey, participants rated their perceived abilities relatively highly, limiting the instrument’s ability to measure improvement on the post-intervention survey.

Our study participants’ attitudes toward palliative care were high in both the pre- and post-survey, suggesting a relatively positive and accepting attitude toward this topic. This may have reflected a high level of interest in the topic and subsequently may have affected motivations to engage in the curriculum. Thus, our results may not generalize to institutions with varying attitudes toward palliative care or to environments where there is heightened resistance toward or barriers to providing this type of curriculum in the ED.

Our short-term assessment period may also not have captured long-term retention or application of knowledge and skills. Incorporating objective assessments, a longitudinal follow-up, or the use of randomized groups would have strengthened our results. Nonetheless, the findings highlight the value of a mixed didactic, interactive, and experiential curriculum in advancing the care of patients with palliative care needs in the ED and ensuring residents are better equipped to manage these complex clinical scenarios. The demand on resources and time is an additional consideration, and this may pose some barriers to others wishing to implement a multimodal curriculum such as this.

## CONCLUSION

Our palliative care curriculum for EM residents significantly improved their self-reported knowledge, level of comfort, and perceived application of skills in caring for patients with chronic and/or terminal illnesses in the ED. They found palliative care education to be important. Lecture, small-group discussion, and simulation were all felt to be effective modalities for learning palliative care skills. Our results highlight the feasibility and effectiveness of embedding structured palliative care training in EM residency training, and the implementation of similar curricula is likely broadly scalable.

Given the frequency with which emergency physicians encounter patients with serious illness, integrating longitudinal approaches may further strengthen residents’ preparedness. Broader adoption of such curricula could help align EM training with emerging national best-practice guidelines and milestones for palliative care in emergency medicine.

## Figures and Tables

**Table 1:** Survey results of emergency medicine residents’ perceived palliative care knowledge and skills pre- versus post-implementation of a palliative care curriculum.

Survey Question	Median Score (Interquartile Range)		Wilcoxon signed-rank *P* value	Effect Size (Cohen d)

	Pre-test N = 41	Postest N = 41		
Emergency medicine has an important role in providing palliative care.	5 (4–5)	5 (4–5)	1	0
Palliative care education is important in emergency medicine residency.	4 (4–5)	4 (4–5)	.83	0.03
I understand the difference between hospice and palliative care.	3 (2–4)	4 (4–5)	< .001*	0.97
I feel confident…				
Determining decision-making capacity	4 (3–4)	4 (4–5)	< .001*	0.73
Having goals-of-care discussions	4 (3–4)	4 (4–5)	< .001*	1.14
Interpreting advance directive forms	3 (2–4)	4 (4–4)	< .001*	1.01
Managing symptoms associated with the last hours of living	3 (2–3)	4 (3–4)	< .0001*	1.25
Negotiating decision-making regarding risks, benefits, and alternatives in patients with chronic or terminal illnesses	3 (2–3)	4 (4–4)	< .001*	1.18
Treating refractory symptoms associated with terminal disease	2 (2–3)	4 (3–4)	< .001*	1.14
Performing acute pain management in patients with palliative care needs	3 (2–4)	4 (4–4)	< .001*	1.09
Communicating bad news including death, errors, and unexpected outcomes while caring for patients with life-limiting illnesses	3 (2–4)	4 (4–4)	< .001*	0.97
Initiating contact and involving palliative and/or hospice care teams	2 (2–3)	4 (4–4)	< .001*	1.12
